# Supplementary material for: Promoter choice: Who should drive the CAR in T cells?
Source: PLoS One. 2020 Jul 24;15(7):e0232915. doi: 10.1371/journal.pone.0232915 (PMC7380635; doi:10.1371/journal.pone.0232915)
Supplement: S1 Raw images — (PDF) [file pone.0232915.s001.pdf]

Fig. 1b

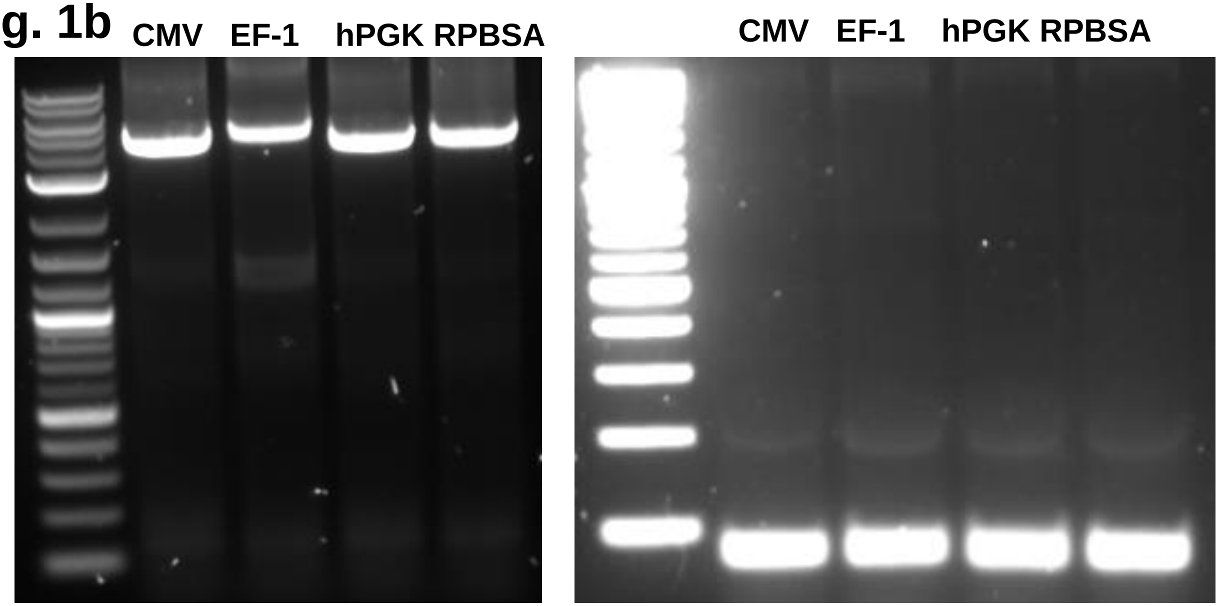

Original Fig. 1b

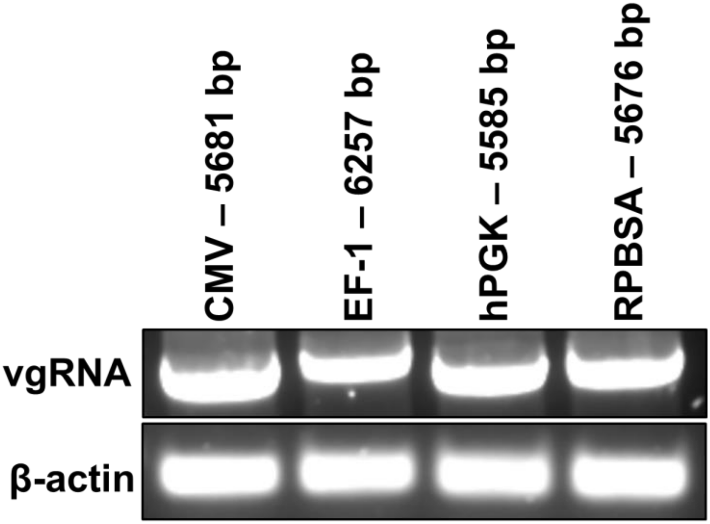

manuscript's Fig. 1b

Fig. 2

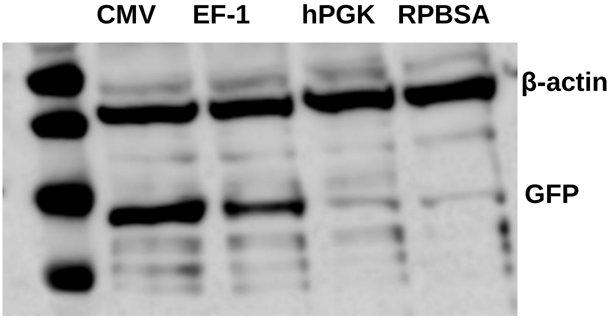

Original Fig. 2a

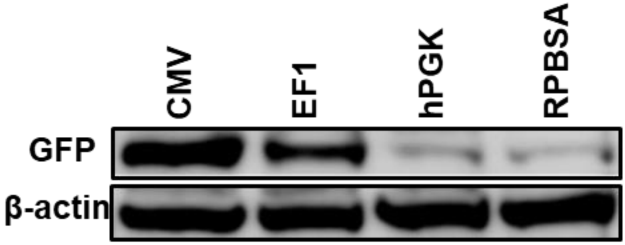

Manuscript- Fig.2a

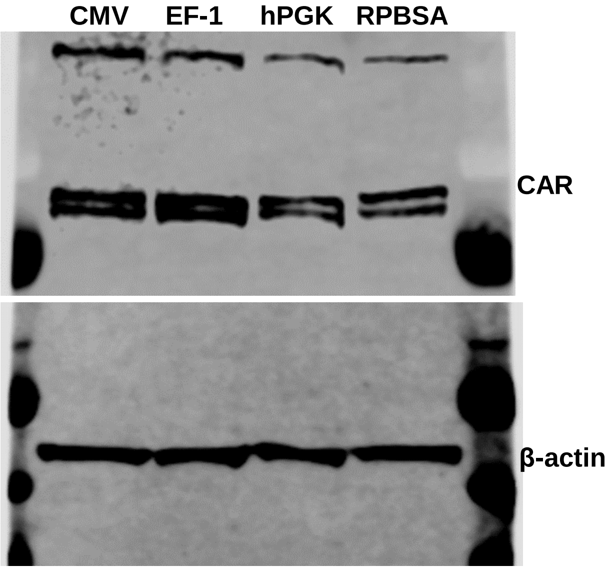

Original Fig. 2b

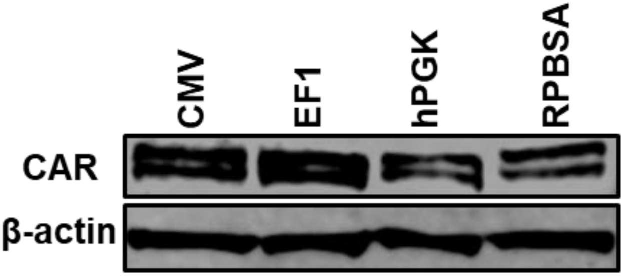

Manuscript- Fig.2b

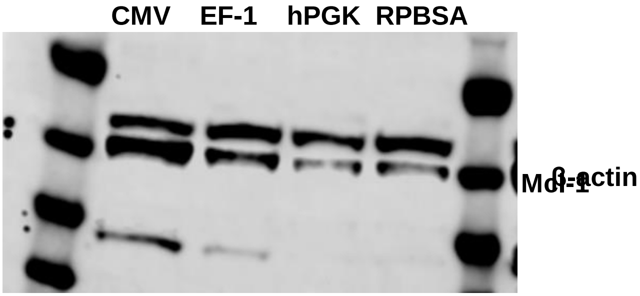

Original Fig. 2c

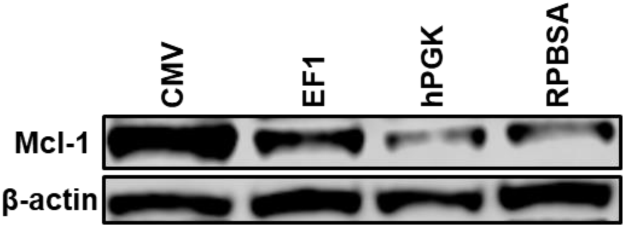

Manuscript- Fig.2c

Fig. 6c

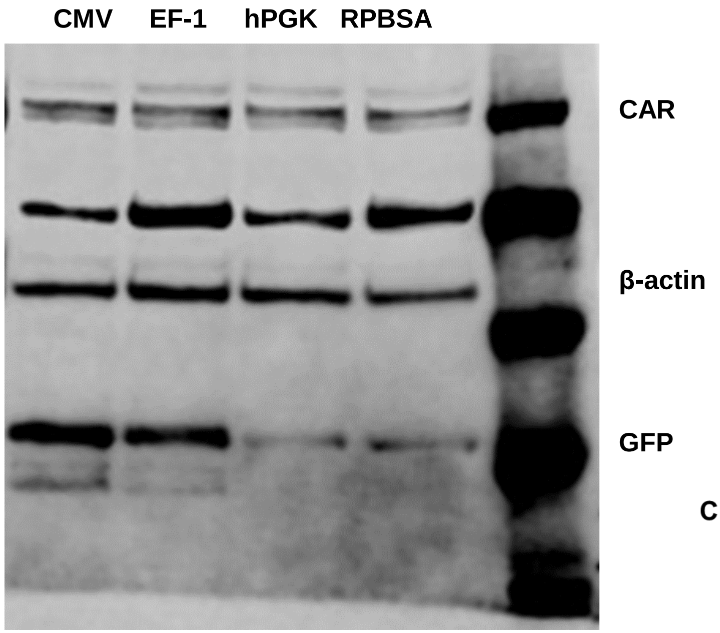

Original Fig. 6c

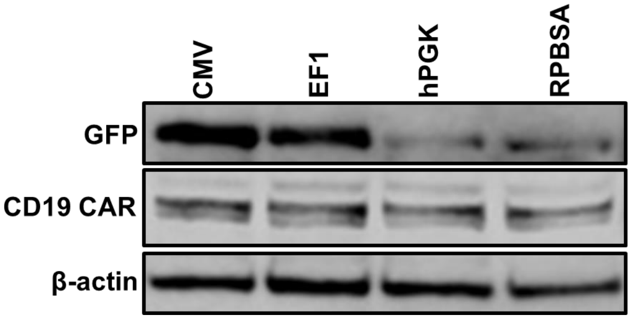

Manuscript- Fig.6c
