# Supplementary material for: Promoter choice: Who should drive the CAR in T cells?
Source: PLoS One. 2020 Jul 24;15(7):e0232915. doi: 10.1371/journal.pone.0232915 (PMC7380635; doi:10.1371/journal.pone.0232915)
Supplement: S1 Data — (DOCX) [file pone.0232915.s002.docx]

**LZ-CD95L production and Resazurin assay**

The pcDNA3.1(-)-LZ-CD95L plasmid containing isoleucine zipped human CD95 ligand (LZ-CD95L) with histidine tag (made in house and available from Addgene #104349) was transfected into Expi293 cells (1.0 µg DNA per mL of culture volume with exp293 transfection reagent; Thermofisher) and LZ-CD95L isolated by nickel chromatography.

The ability of LZ-CD95L to induce activation-induced cell death (AICD) was determined using the resazurin assay. Jurkat cells (50 × 10^3^ seeded in 96-well plate in 100 µl) were treated with variety of concentrations 0-1.5 µg/ ml) LZ-CD95L. After 24 hours, 10 µl of resazurin solution (12 mg/L of resazurin, 10 mg/ L methylene blue, 40 μM potassium ferricyanide, 40 μM potassium ferrocyanide and 100 mM phosphate buffer pH 7.4) was added to the wells. After a further 4 hours culture, fluorescence (ex540 nm / em585 nm) was determined using Varioskan LUX multimode microplate reader (Thermo Fisher, USA).


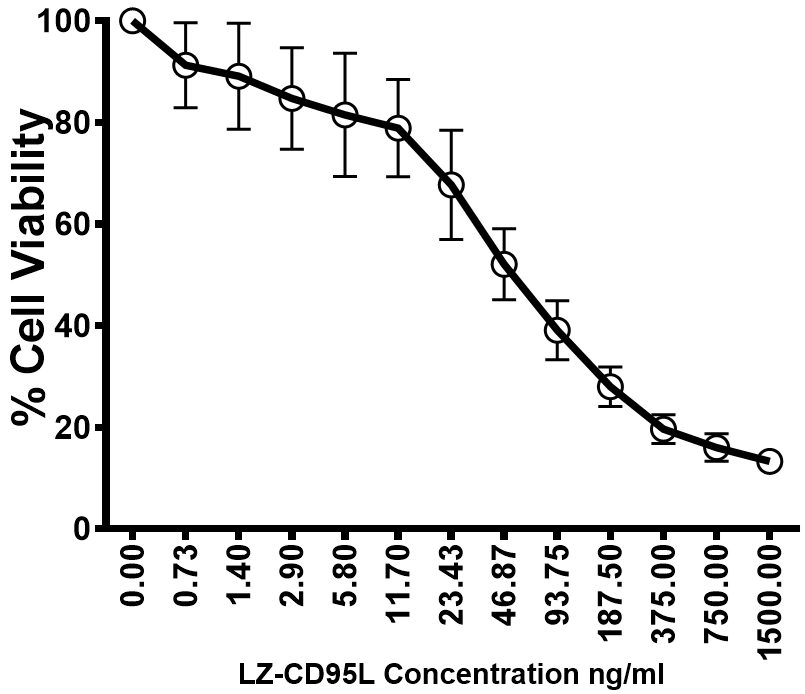


S 1. Ability of LZ-CD95L in inducing AICD in Jurkat cells.

**CMV promoter sequence**

ACATTGATTATTGACTAGTTATTAATAGTAATCAATTACGGGGTCATTAG 50

TTCATAGCCCATATATGGAGTTCCGCGTTACATAACTTACGGTAAATGGC 100

CCGCCTGGCTGACCGCCCAACGACCCCCGCCCATTGACGTCAATAATGAC 150

GTATGTTCCCATAGTAACGCCAATAGGGACTTTCCATTGACGTCAATGGG 200

TGGAGTATTTACGGTAAACTGCCCACTTGGCAGTACATCAAGTGTATCAT 250

ATGCCAAGTACGCCCCCTATTGACGTCAATGACGGTAAATGGCCCGCCTG 300

GCATTATGCCCAGTACATGACCTTATGGGACTTTCCTACTTGGCAGTACA 350

TCTACGTATTAGTCATCGCTATTACCATGGTGATGCGGTTTTGGCAGTAC 400

ATCAATGGGCGTGGATAGCGGTTTGACTCACGGGGATTTCCAAGTCTCCA 450

CCCCATTGACGTCAATGGGAGTTTGTTTTGGCACCAAAATCAACGGGACT 500

TTCCAAAATGTCGTAACAACTCCGCCCCATTGACGCAAATGGGCGGTAGG 550

CGTGTACGGTGGGAGGTCTATATAAGCAGAGCTCTCTGGCTAACTAGAGA 600

ACCCACTGCTTACTGGC 617

**EF1 promoter sequence**

AAGCTTGATATCGGGCTCCGGTGCCCGTCAGTGGGCAGAGCGCACATCGC 50

CCACAGTCCCCGAGAAGTTGGGGGGAGGGGTCGGCAATTGAACCGGTGCC 100

TAGAGAAGGTGGCGCGGGGTAAACTGGGAAAGTGATGTCGTGTACTGGCT 150

CCGCCTTTTTCCCGAGGGTGGGGGAGAACCGTATATAAGTGCAGTAGTCG 200

CCGTGAACGTTCTTTTTCGCAACGGGTTTGCCGCCAGAACACAGGTAAGT 250

GCCGTGTGTGGTTCCCGCGGGCCTGGCCTCTTTACGGGTTATGGCCCTTG 300

CGTGCCTTGAATTACTTCCACGCCCCTGGCTGCAGTACGTGATTCTTGAT 350

CCCGAGCTTCGGGTTGGAAGTGGGTGGGAGAGTTCGAGGCCTTGCGCTTA 400

AGGAGCCCCTTCGCCTCGTGCTTGAGTTGAGGCTTGGCCTGGGCGCTGGG 450

GCCGCCGCGTGCGAATCTGGTGGCACCTTCGCGCCTGTCTCGCTGCTTTC 500

GATAAGTCTCTAGCCATTTAAAATTTTTGATGACCTGCTGCGACGCTTTT 550

TTTCTGGCAAGATAGTCTTGTAAATGCGGGCCAAGATCTGCACACTGGTA 600

TTTCGGTTTTTGGGGCCGCGGGCGGCGACGGGGCCCGTGCGTCCCAGCGC 650

ACATGTTCGGCGAGGCGGGGCCTGCGAGCGCGGCCACCGAGAATCGGACG 700

GGGGTAGTCTCAAGCTGGCCGGCCTGCTCTGGTGCCTGGCCTCGCGCCGC 750

CGTGTATCGCCCCGCCCTGGGCGGCAAGGCTGGCCCGGTCGGCACCAGTT 800

GCGTGAGCGGAAAGATGGCCGCTTCCCGGCCCTGCTGCAGGGAGCTCAAA 850

ATGGAGGACGCGGCGCTCGGGAGAGCGGGCGGGTGAGTCACCCACACAAA 900

GGAAAAGGGCCTTTCCGTCCTCAGCCGTCGCTTCATGTGACTCCACGGAG 950

TACCGGGCGCCGTCCAGGCACCTCGATTAGTTCTCGAGCTTTTGGAGTAC 1000

GTCGTCTTTAGGTTGGGGGGAGGGGTTTTATGCGATGGAGTTTCCCCATA 1050

CTGAGTGGGTGGAGACTGAAGTTAGGCCAGCTTGGCACTTGATGTAATTC 1100

TCCTTGGAATTTGCCCTTTTTGAGTTTGGATCTTGGTTCATTCTCAAGCC 1150

TCAGACAGTGGTTCAAAGTTTTTTTCTTCCATTTAAGGTGTCGTGAAAAC 1200

TACCCCGGATCCGTGGT 1217

**hPGK promoter sequence**

CCACGGGGTTGGGGTTGCGCCTTTTCCAAGGCAGCCCTGGGTTTGCGCAG 50

GGACGCGGCTGCTCTGGGCGTGGTTCCGGGAAACGCAGCGGCGCCGACCC 100

TGGGTCTCGCACATTCTTCACGTCCGTTCGCAGCGTCACCCGGATCTTCG 150

CCGCTACCCTTGTGGGCCCCCCGGCGACGCTTCCTGCTCCGCCCCTAAGT 200

CGGGAAGGTTCCTTGCGGTTCGCGGCGTGCCGGACGTGACAAACGGAAGC 250

CGCACGTCTCACTAGTACCCTCGCAGACGGACAGCGCCAGGGAGCAATGG 300

CAGCGCGCCGACCGCGATGGGCTGTGGCCAATAGCGGCTGCTCAGCGGGG 350

CGCGCCGAGAGCAGCGGCCGGGAAGGGGCGGTGCGGGAGGCGGGGTGTGG 400

GGCGGTAGTGTGGGCCCTGTTCCTGCCCGCGCGGTGTTCCGCATTCTGCA 450

AGCCTCCGGAGCGCACGTCGGCAGTCGGCTCCCTCGTTGACCGAATCACC 500

GACCTCTCTCCCCAGG 516

**RPBSA promoter sequence**

AAGCTTGATATCGCGAGACCCTGTCTCACAAAATAAAGTAAGCCCGGACT 50

GAGTGCGGAAAGGCGGGCCTGGCGGGTCTGGTCTCCCCATGCGGGCCACC 100

AGAGGCCCTGCAGCCTTCAGTCGCTTGAAGGGGTAATGGCGCTTCCACTC 150

ACAAACATGGCGGACAGAGCGTGTGAACGAGATGAACAGCCCCTCAAAAA 200

TATGGCCGCCGAGGCTGGACGGCCGTGCCCCAGCAGCACCGCCTCCGCGC 250

CCCACGTGATCTCTCGCCGGGCACAGCGCTGACCGCGGAGGTCCAACCGG 300

AAGAATGTCCGGATTGGACATTCGGAAGAGGGCCCGCCTTCCCTGGGGAA 350

TCTCTGCGCACGCGCAGAACGCTTCGACCAATGAAAACACAGGAAGCCGT 400

CCGCGCAACCGCGTTGCGTCACTTCTGCCGCCCCTGTTTCAAGGTATATA 450

GCCGTAGACGGAACTTCGCCTTTCTCTCGGCCTTAGCGCCATTTTTTTGG 500

GTGAGTGTTTTTTGGTTCCTGCGTTGGGATTCCGTGTACAATCCATAGAC 550

ATCTGACCTCGGCACTTAGCATCATCACAGCAAACTAACTGTAGCCTTTC 600

TCTCTTTCCCTGTAGAAACCTCTGCGGATCCGTGGT 636

**Table. 1 Core promoter element predicted for CMV promoter**

| **Motif** | **Pos** | **Score** | **Sequence** |
| --- | --- | --- | --- |
| **INR** | 310 | 0.96 | CCAGTAC |
| **INR** | 223 | 0.95 | CCACTTG |
| **INR** | 170 | 0.91 | CCAATAG |
| **INR** | 44 | 0.88 | TCATTAG |
| **INR** | 47 | 0.85 | TTAGTTC |
| **INR** | 369 | 0.84 | CTATTAC |
| **INR** | 503 | 0.83 | CCAAAAT |
| **INR** | 484 | 0.83 | CCAAAAT |
| **INR** | 336 | 0.82 | CTACTTG |
| **INR** | 247 | 0.82 | TCATATG |
| **TATA** | 567 | 0.93 | TCTATATAAGCA |
| **TATA** | 58 | 0.85 | CCCATATATGGA |
| **DPE** | 413 | 0.92 | GGATA |
| **DPE** | 361 | 0.91 | AGTCA |
| **DPE** | 42 | 0.90 | GGTCA |
| **DPE** | 69 | 0.89 | AGTTC |
| **DPE** | 49 | 0.89 | AGTTC |
| **DPE** | 17 | 0.88 | AGTTA |

| **Motif** | **Pos** | **Score** | **Sequence** |
| --- | --- | --- | --- |
| **INR** | 782 | 0.95 | CCAGTTG |
| **INR** | 1125 | 0.94 | TCATTCT |
| **INR** | 1033 | 0.87 | CCATACT |
| **INR** | 221 | 0.86 | CCAGAAC |
| **INR** | 299 | 0.85 | TTACTTC |
| **INR** | 964 | 0.85 | TTAGTTC |
| **INR** | 833 | 0.81 | TCAAAAT |
| **TATA** | 167 | 0.92 | CGTATATAAGTG |
| **TATA** | 502 | 0.84 | CATTTAAAATTT |
| **MTE** | 698 | 0.87 | CAAGCTGGCCGG |
| **MTE** | 662 | 0.81 | CGAGCGCGGCCA |
| **DPE** | 843 | 0.99 | GGACG |
| **DPE** | 683 | 0.99 | GGACG |
| **DPE** | 800 | 0.97 | AGATG |
| **DPE** | 1140 | 0.96 | AGACA |
| **DPE** | 183 | 0.95 | AGTCG |
| **DPE** | 571 | 0.94 | AGATC |
| **DPE** | 774 | 0.94 | GGTCG |
| **DPE** | 66 | 0.94 | GGTCG |
| **DPE** | 1115 | 0.93 | GGATC |
| **DPE** | 547 | 0.93 | AGATA |
| **DPE** | 784 | 0.92 | AGTTG |
| **DPE** | 53 | 0.92 | AGTTG |
| **DPE** | 412 | 0.92 | AGTTG |
| **DPE** | 42 | 0.92 | AGTCC |
| **DPE** | 998 | 0.91 | GGTTG |
| **DPE** | 349 | 0.91 | GGTTG |
| **DPE** | 873 | 0.91 | AGTCA |
| **DPE** | 966 | 0.89 | AGTTC |
| **DPE** | 368 | 0.89 | AGTTC |
| **DPE** | 247 | 0.89 | GGTTC |
| **DPE** | 1122 | 0.89 | GGTTC |
| **DPE** | 1147 | 0.89 | GGTTC |
| **DPE** | 1057 | 0.88 | AGTTA |
| **DPE** | 274 | 0.87 | GGTTA |
| **BRE** | 305 | 0.97 | CCACGCC |
| **BRE** | 942 | 0.91 | GGGCGCC |

**Table. 2 Core promoter element predicted for EF1 promoter**

**Table. 3 Core promoter element predicted for hPGK promoter**

| **Motif** | **Pos** | **Score** | **Sequence** |
| --- | --- | --- | --- |
| **INR** | 328 | 0.91 | CCAATAG |
| **INR** | 259 | 0.89 | TCACTAG |
| **INR** | 262 | 0.84 | CTAGTAC |
| **MTE** | 300 | 0.93 | GCAGCGCGCCGA |
| **MTE** | 80 | 0.90 | GAAACGCAGCGG |
| **DPE** | 275 | 1.00 | AGACG |
| **DPE** | 51 | 0.99 | GGACG |
| **DPE** | 232 | 0.99 | GGACG |
| **DPE** | 198 | 0.95 | AGTCG |
| **DPE** | 473 | 0.95 | AGTCG |
| **DPE** | 279 | 0.95 | GGACA |
| **DPE** | 142 | 0.93 | GGATC |
| **DPE** | 7 | 0.91 | GGTTG |
| **DPE** | 13 | 0.91 | GGTTG |
| **DPE** | 207 | 0.89 | GGTTC |
| **DPE** | 72 | 0.89 | GGTTC |
| **DPE** | 217 | 0.89 | GGTTC |
| **BRE** | 89 | 0.96 | CGGCGCC |
| **BRE** | 350 | 0.96 | GCGCGCC |
| **BRE** | 303 | 0.96 | GCGCGCC |

**Table. 4 Core promoter element predicted for RPBSA promoter**

| **Motif** | **Pos** | **Score** | **Sequence** |
| --- | --- | --- | --- |
| **INR** | 406 | 0.97 | TCACTTC |
| **INR** | 476 | 0.97 | CCATTTT |
| **INR** | 104 | 0.92 | TCAGTCG |
| **TATA** | 430 | 0.88 | GGTATATAGCCG |
| **TATA** | 17 | 0.86 | AAAATAAAGTAA |
| **DPE** | 443 | 1.00 | AGACG |
| **DPE** | 204 | 0.99 | GGACG |
| **DPE** | 167 | 0.97 | AGATG |
| **DPE** | 3 | 0.97 | AGACC |
| **DPE** | 534 | 0.96 | AGACA |
| **DPE** | 106 | 0.95 | AGTCG |
| **DPE** | 303 | 0.95 | GGACA |
| **DPE** | 149 | 0.95 | GGACA |
| **DPE** | 277 | 0.91 | GGTCC |
| **DPE** | 501 | 0.89 | GGTTC |
| **BRE** | 232 | 1.00 | CCGCGCC |

**Table. 5 The TF binding sites predicted by AliBaba program for CMV promoter.**

**Promoter: CMV**

**Length: 617 bp**

**Number of sites found: 97**

Class Factor Start Stop

---------------------------------------------------

[4.3.1.1](http://transfac.gbf.de/TRANSFAC/cl/cl.html#4.3.1.1) MEB-1 17 26

[1.2.8.0](http://transfac.gbf.de/TRANSFAC/cl/cl.html#1.2.8.0) Id3 29 38

[3.1.1.12](http://transfac.gbf.de/TRANSFAC/cl/cl.html#3.1.1.12) HNF-1C 29 38

[4.1.3.0](http://transfac.gbf.de/TRANSFAC/cl/cl.html#4.1.3.0) NF-ATc3 29 38

[9.9.29](http://transfac.gbf.de/TRANSFAC/cl/cl.html#9.9.29) AP-1 37 46

[2.1.2.3](http://transfac.gbf.de/TRANSFAC/cl/cl.html#2.1.2.3) T3R 38 47

[4.3.2.0](http://transfac.gbf.de/TRANSFAC/cl/cl.html#4.3.2.0) SRF 58 68

[1.1.3.0](http://transfac.gbf.de/TRANSFAC/cl/cl.html#1.1.3.0) C/EBPalpha 75 84

[2.3.1.0](http://transfac.gbf.de/TRANSFAC/cl/cl.html#2.3.1.0) Sp1 96 108

[1.1.1.1](http://transfac.gbf.de/TRANSFAC/cl/cl.html#1.1.1.1) c-Jun 103 112

[2.3.1.0](http://transfac.gbf.de/TRANSFAC/cl/cl.html#2.3.1.0) Sp1 109 118

[2.3.1.0](http://transfac.gbf.de/TRANSFAC/cl/cl.html#2.3.1.0) Sp1 122 134

[2.3.2.1](http://transfac.gbf.de/TRANSFAC/cl/cl.html#2.3.2.1) Egr-1 123 132

[1.1.1.6](http://transfac.gbf.de/TRANSFAC/cl/cl.html#1.1.1.6) CRE-BP1 131 140

[1.1.2.0](http://transfac.gbf.de/TRANSFAC/cl/cl.html#1.1.2.0) CREB 131 140

[2.3.3.0](http://transfac.gbf.de/TRANSFAC/cl/cl.html#2.3.3.0) CPE_binding_pro 131 140

[9.9.51](http://transfac.gbf.de/TRANSFAC/cl/cl.html#9.9.51) ATF 131 140

[1.1.1.6](http://transfac.gbf.de/TRANSFAC/cl/cl.html#1.1.1.6) CRE-BP1 142 151

[2.3.3.0](http://transfac.gbf.de/TRANSFAC/cl/cl.html#2.3.3.0) CPE_binding_pro 143 152

[9.9.51](http://transfac.gbf.de/TRANSFAC/cl/cl.html#9.9.51) ATF 144 153

[4.3.2.0](http://transfac.gbf.de/TRANSFAC/cl/cl.html#4.3.2.0) SRF 169 178

[2.3.4.0](http://transfac.gbf.de/TRANSFAC/cl/cl.html#2.3.4.0) MBP-1_(1) 175 184

[9.9.588](http://transfac.gbf.de/TRANSFAC/cl/cl.html#9.9.588) NF-kappaB 175 184

[9.9.591](http://transfac.gbf.de/TRANSFAC/cl/cl.html#9.9.591) NF-kappaB(-like 175 184

[4.1.1.0](http://transfac.gbf.de/TRANSFAC/cl/cl.html#4.1.1.0) c-Rel 176 185

[9.9.590](http://transfac.gbf.de/TRANSFAC/cl/cl.html#9.9.590) NF-kappaB 176 185

[1.1.1.6](http://transfac.gbf.de/TRANSFAC/cl/cl.html#1.1.1.6) CRE-BP1 184 193

[1.1.2.0](http://transfac.gbf.de/TRANSFAC/cl/cl.html#1.1.2.0) CREB 184 193

[2.3.3.0](http://transfac.gbf.de/TRANSFAC/cl/cl.html#2.3.3.0) CPE_binding_pro 184 193

[9.9.51](http://transfac.gbf.de/TRANSFAC/cl/cl.html#9.9.51) ATF 184 193

[2.3.1.0](http://transfac.gbf.de/TRANSFAC/cl/cl.html#2.3.1.0) Sp1 193 205

[9.9.77](http://transfac.gbf.de/TRANSFAC/cl/cl.html#9.9.77) CACCC-binding_f 195 204

[2.3.1.0](http://transfac.gbf.de/TRANSFAC/cl/cl.html#2.3.1.0) Sp1 217 230

[9.9.539](http://transfac.gbf.de/TRANSFAC/cl/cl.html#9.9.539) NF-1 224 233

[2.2.1.1](http://transfac.gbf.de/TRANSFAC/cl/cl.html#2.2.1.1) GATA-1 244 253

[3.1.2.2](http://transfac.gbf.de/TRANSFAC/cl/cl.html#3.1.2.2) Oct-1 244 255

[9.9.539](http://transfac.gbf.de/TRANSFAC/cl/cl.html#9.9.539) NF-1 249 260

[1.1.1.6](http://transfac.gbf.de/TRANSFAC/cl/cl.html#1.1.1.6) CRE-BP1 267 276

[1.1.2.0](http://transfac.gbf.de/TRANSFAC/cl/cl.html#1.1.2.0) CREB 267 276

[2.3.1.0](http://transfac.gbf.de/TRANSFAC/cl/cl.html#2.3.1.0) YY1 289 301

[2.3.1.0](http://transfac.gbf.de/TRANSFAC/cl/cl.html#2.3.1.0) Sp1 307 316

[2.1.2.1](http://transfac.gbf.de/TRANSFAC/cl/cl.html#2.1.2.1) RAR-alpha1 315 324

[2.1.2.3](http://transfac.gbf.de/TRANSFAC/cl/cl.html#2.1.2.3) T3R-alpha 315 325

[2.1.1.4](http://transfac.gbf.de/TRANSFAC/cl/cl.html#2.1.1.4) ER 316 325

[2.2.2.0](http://transfac.gbf.de/TRANSFAC/cl/cl.html#2.2.2.0) Ttx 316 325

[2.3.4.0](http://transfac.gbf.de/TRANSFAC/cl/cl.html#2.3.4.0) KBP-1 326 335

[4.1.1.0](http://transfac.gbf.de/TRANSFAC/cl/cl.html#4.1.1.0) c-Rel 326 335

[9.9.213](http://transfac.gbf.de/TRANSFAC/cl/cl.html#9.9.213) EBP-1 326 335

[9.9.588](http://transfac.gbf.de/TRANSFAC/cl/cl.html#9.9.588) NF-kappaB 326 335

[9.9.590](http://transfac.gbf.de/TRANSFAC/cl/cl.html#9.9.590) NF-kappaB 326 335

[9.9.591](http://transfac.gbf.de/TRANSFAC/cl/cl.html#9.9.591) NF-kappaB(-like 326 335

[9.9.539](http://transfac.gbf.de/TRANSFAC/cl/cl.html#9.9.539) NF-1 337 346

[1.1.1.2](http://transfac.gbf.de/TRANSFAC/cl/cl.html#1.1.1.2) c-Fos 355 366

[1.1.1.5](http://transfac.gbf.de/TRANSFAC/cl/cl.html#1.1.1.5) GCN4 356 365

[1.1.1.1](http://transfac.gbf.de/TRANSFAC/cl/cl.html#1.1.1.1) c-Jun 357 366

[9.9.29](http://transfac.gbf.de/TRANSFAC/cl/cl.html#9.9.29) AP-1 357 366

[1.1.3.0](http://transfac.gbf.de/TRANSFAC/cl/cl.html#1.1.3.0) C/EBPalpha(p20) 369 378

[9.9.539](http://transfac.gbf.de/TRANSFAC/cl/cl.html#9.9.539) NF-1 386 395

[2.3.1.0](http://transfac.gbf.de/TRANSFAC/cl/cl.html#2.3.1.0) Sp1 403 413

[1.1.1.1](http://transfac.gbf.de/TRANSFAC/cl/cl.html#1.1.1.1) c-Jun 420 429

[1.1.1.2](http://transfac.gbf.de/TRANSFAC/cl/cl.html#1.1.1.2) c-Fos 420 429

[9.9.29](http://transfac.gbf.de/TRANSFAC/cl/cl.html#9.9.29) AP-1 423 432

[2.3.4.0](http://transfac.gbf.de/TRANSFAC/cl/cl.html#2.3.4.0) AGIE-BP1 431 440

[9.9.213](http://transfac.gbf.de/TRANSFAC/cl/cl.html#9.9.213) EBP-1 431 440

[9.9.588](http://transfac.gbf.de/TRANSFAC/cl/cl.html#9.9.588) NF-kappaB 431 440

[9.9.590](http://transfac.gbf.de/TRANSFAC/cl/cl.html#9.9.590) NF-kappaB 431 440

[9.9.594](http://transfac.gbf.de/TRANSFAC/cl/cl.html#9.9.594) RelA 431 440

[4.1.1.0](http://transfac.gbf.de/TRANSFAC/cl/cl.html#4.1.1.0) NF-kappaB 432 441

[1.1.3.0](http://transfac.gbf.de/TRANSFAC/cl/cl.html#1.1.3.0) C/EBPalpha 434 443

[9.9.537](http://transfac.gbf.de/TRANSFAC/cl/cl.html#9.9.537) NF-1 437 446

[2.3.1.0](http://transfac.gbf.de/TRANSFAC/cl/cl.html#2.3.1.0) Sp1 444 456

[1.1.1.6](http://transfac.gbf.de/TRANSFAC/cl/cl.html#1.1.1.6) CRE-BP1 453 462

[1.1.2.0](http://transfac.gbf.de/TRANSFAC/cl/cl.html#1.1.2.0) CREB 453 462

[2.3.3.0](http://transfac.gbf.de/TRANSFAC/cl/cl.html#2.3.3.0) CPE_binding_pro 453 462

[9.9.51](http://transfac.gbf.de/TRANSFAC/cl/cl.html#9.9.51) ATF 453 462

[9.9.539](http://transfac.gbf.de/TRANSFAC/cl/cl.html#9.9.539) NF-1 474 483

[2.3.4.0](http://transfac.gbf.de/TRANSFAC/cl/cl.html#2.3.4.0) MBP-1_(1) 494 503

[4.1.1.0](http://transfac.gbf.de/TRANSFAC/cl/cl.html#4.1.1.0) c-Rel 494 503

[9.9.588](http://transfac.gbf.de/TRANSFAC/cl/cl.html#9.9.588) NF-kappaB 494 503

[9.9.590](http://transfac.gbf.de/TRANSFAC/cl/cl.html#9.9.590) NF-kappaB 494 503

[9.9.591](http://transfac.gbf.de/TRANSFAC/cl/cl.html#9.9.591) NF-kappaB(-like 494 503

[9.9.594](http://transfac.gbf.de/TRANSFAC/cl/cl.html#9.9.594) RelA 494 503

[1.1.3.0](http://transfac.gbf.de/TRANSFAC/cl/cl.html#1.1.3.0) C/EBPalpha 497 506

[9.9.1469](http://transfac.gbf.de/TRANSFAC/cl/cl.html#9.9.1469) Ik-1 498 507

[9.9.1470](http://transfac.gbf.de/TRANSFAC/cl/cl.html#9.9.1470) Ik-2 498 507

[9.9.1471](http://transfac.gbf.de/TRANSFAC/cl/cl.html#9.9.1471) Ik-3 498 507

[9.9.535](http://transfac.gbf.de/TRANSFAC/cl/cl.html#9.9.535) NF-1 500 509

[9.9.539](http://transfac.gbf.de/TRANSFAC/cl/cl.html#9.9.539) NF-1 500 509

[2.3.1.0](http://transfac.gbf.de/TRANSFAC/cl/cl.html#2.3.1.0) YY1 503 512

[9.9.51](http://transfac.gbf.de/TRANSFAC/cl/cl.html#9.9.51) ATF 505 514

[2.3.1.0](http://transfac.gbf.de/TRANSFAC/cl/cl.html#2.3.1.0) Sp1 517 527

[9.9.51](http://transfac.gbf.de/TRANSFAC/cl/cl.html#9.9.51) ATF 525 534

[1.1.3.0](http://transfac.gbf.de/TRANSFAC/cl/cl.html#1.1.3.0) C/EBPalpha 528 537

[2.3.1.0](http://transfac.gbf.de/TRANSFAC/cl/cl.html#2.3.1.0) Sp1 536 545

[2.3.1.0](http://transfac.gbf.de/TRANSFAC/cl/cl.html#2.3.1.0) Sp1 555 565

[4.3.2.0](http://transfac.gbf.de/TRANSFAC/cl/cl.html#4.3.2.0) SRF 566 575

[1.1.1.1](http://transfac.gbf.de/TRANSFAC/cl/cl.html#1.1.1.1) c-Jun 585 594

**Table. 6 The TF binding sites predicted by AliBaba program for EF-1 promoter.**

**Promoter: EF1**

**Length: 1192 bp**

**Number of sites found: 109**

Class Factor Start Stop

---------------------------------------------------

[9.9.51](http://transfac.gbf.de/TRANSFAC/cl/cl.html#9.9.51) ATF 12 21

[2.3.1.0](http://transfac.gbf.de/TRANSFAC/cl/cl.html#2.3.1.0) Sp1 15 27

[1.1.3.0](http://transfac.gbf.de/TRANSFAC/cl/cl.html#1.1.3.0) C/EBPalpha 30 39

[2.3.1.0](http://transfac.gbf.de/TRANSFAC/cl/cl.html#2.3.1.0) Sp1 38 47

[2.3.3.0](http://transfac.gbf.de/TRANSFAC/cl/cl.html#2.3.3.0) MIG1 55 64

[2.3.1.0](http://transfac.gbf.de/TRANSFAC/cl/cl.html#2.3.1.0) Sp1 56 70

[2.3.1.0](http://transfac.gbf.de/TRANSFAC/cl/cl.html#2.3.1.0) Sp1 94 106

[1.3.1.2](http://transfac.gbf.de/TRANSFAC/cl/cl.html#1.3.1.2) USF 97 106

[9.9.701](http://transfac.gbf.de/TRANSFAC/cl/cl.html#9.9.701) PTF1-beta 111 120

[9.9.428](http://transfac.gbf.de/TRANSFAC/cl/cl.html#9.9.428) ISGF-3 112 121

[2.3.1.0](http://transfac.gbf.de/TRANSFAC/cl/cl.html#2.3.1.0) Sp1 132 143

[9.9.77](http://transfac.gbf.de/TRANSFAC/cl/cl.html#9.9.77) CACCC-binding_f 150 159

[2.3.1.0](http://transfac.gbf.de/TRANSFAC/cl/cl.html#2.3.1.0) Sp1 150 162

[2.3.3.0](http://transfac.gbf.de/TRANSFAC/cl/cl.html#2.3.3.0) MIG1 154 163

[9.9.535](http://transfac.gbf.de/TRANSFAC/cl/cl.html#9.9.535) NF-1 199 208

[1.3.2.3](http://transfac.gbf.de/TRANSFAC/cl/cl.html#1.3.2.3) E2F 213 222

[9.9.726](http://transfac.gbf.de/TRANSFAC/cl/cl.html#9.9.726) repressor_of_CA 214 223

[2.1.1.1](http://transfac.gbf.de/TRANSFAC/cl/cl.html#2.1.1.1) GR 221 230

[2.3.1.0](http://transfac.gbf.de/TRANSFAC/cl/cl.html#2.3.1.0) Sp1 250 264

[2.3.1.0](http://transfac.gbf.de/TRANSFAC/cl/cl.html#2.3.1.0) Sp1 257 266

[4.3.2.0](http://transfac.gbf.de/TRANSFAC/cl/cl.html#4.3.2.0) SRF 275 284

[1.1.3.0](http://transfac.gbf.de/TRANSFAC/cl/cl.html#1.1.3.0) C/EBPalpha 280 289

[1.2.8.0](http://transfac.gbf.de/TRANSFAC/cl/cl.html#1.2.8.0) Id3 297 306

[3.1.1.12](http://transfac.gbf.de/TRANSFAC/cl/cl.html#3.1.1.12) HNF-1C 297 306

[4.1.3.0](http://transfac.gbf.de/TRANSFAC/cl/cl.html#4.1.3.0) NF-ATc3 297 306

[2.3.1.0](http://transfac.gbf.de/TRANSFAC/cl/cl.html#2.3.1.0) Sp1 303 316

[9.9.539](http://transfac.gbf.de/TRANSFAC/cl/cl.html#9.9.539) NF-1 310 319

[2.3.1.0](http://transfac.gbf.de/TRANSFAC/cl/cl.html#2.3.1.0) Sp1 354 364

[2.3.1.0](http://transfac.gbf.de/TRANSFAC/cl/cl.html#2.3.1.0) Sp1 392 403

[1.6.1.0](http://transfac.gbf.de/TRANSFAC/cl/cl.html#1.6.1.0) AP-2alphaA 398 407

[2.3.1.0](http://transfac.gbf.de/TRANSFAC/cl/cl.html#2.3.1.0) Sp1 414 423

[9.9.539](http://transfac.gbf.de/TRANSFAC/cl/cl.html#9.9.539) NF-1 420 429

[2.3.1.0](http://transfac.gbf.de/TRANSFAC/cl/cl.html#2.3.1.0) Sp1 421 431

[2.3.1.0](http://transfac.gbf.de/TRANSFAC/cl/cl.html#2.3.1.0) Sp1 430 443

[2.3.1.0](http://transfac.gbf.de/TRANSFAC/cl/cl.html#2.3.1.0) Sp1 437 446

[3.1.1.2](http://transfac.gbf.de/TRANSFAC/cl/cl.html#3.1.1.2) Ubx 497 506

[2.3.1.0](http://transfac.gbf.de/TRANSFAC/cl/cl.html#2.3.1.0) Sp1 500 509

[3.1.2.1](http://transfac.gbf.de/TRANSFAC/cl/cl.html#3.1.2.1) Pit-1a 502 511

[2.1.1.4](http://transfac.gbf.de/TRANSFAC/cl/cl.html#2.1.1.4) ER 514 523

[1.1.1.5](http://transfac.gbf.de/TRANSFAC/cl/cl.html#1.1.1.5) GCN4 532 541

[9.9.535](http://transfac.gbf.de/TRANSFAC/cl/cl.html#9.9.535) NF-1 537 546

[1.1.3.0](http://transfac.gbf.de/TRANSFAC/cl/cl.html#1.1.3.0) C/EBPalpha 551 560

[9.9.539](http://transfac.gbf.de/TRANSFAC/cl/cl.html#9.9.539) NF-1 561 572

[2.3.1.0](http://transfac.gbf.de/TRANSFAC/cl/cl.html#2.3.1.0) Sp1 562 571

[9.9.537](http://transfac.gbf.de/TRANSFAC/cl/cl.html#9.9.537) NF-1 562 571

[2.3.1.0](http://transfac.gbf.de/TRANSFAC/cl/cl.html#2.3.1.0) Sp1 571 580

[3.5.3.0](http://transfac.gbf.de/TRANSFAC/cl/cl.html#3.5.3.0) IRF-1 587 596

[2.1.1.1](http://transfac.gbf.de/TRANSFAC/cl/cl.html#2.1.1.1) GR 589 598

[2.3.1.0](http://transfac.gbf.de/TRANSFAC/cl/cl.html#2.3.1.0) Sp1 598 610

[1.6.1.0](http://transfac.gbf.de/TRANSFAC/cl/cl.html#1.6.1.0) AP-2alphaA 599 608

[2.3.1.0](http://transfac.gbf.de/TRANSFAC/cl/cl.html#2.3.1.0) Sp1 604 613

[2.3.1.0](http://transfac.gbf.de/TRANSFAC/cl/cl.html#2.3.1.0) Sp1 611 620

[2.3.1.0](http://transfac.gbf.de/TRANSFAC/cl/cl.html#2.3.1.0) Sp1 617 626

[9.9.270](http://transfac.gbf.de/TRANSFAC/cl/cl.html#9.9.270) ETF 645 654

[2.3.1.0](http://transfac.gbf.de/TRANSFAC/cl/cl.html#2.3.1.0) Sp1 647 658

[2.3.1.0](http://transfac.gbf.de/TRANSFAC/cl/cl.html#2.3.1.0) Sp1 666 675

[2.3.1.0](http://transfac.gbf.de/TRANSFAC/cl/cl.html#2.3.1.0) Sp1 681 690

[2.3.1.0](http://transfac.gbf.de/TRANSFAC/cl/cl.html#2.3.1.0) Sp1 697 709

[2.3.1.0](http://transfac.gbf.de/TRANSFAC/cl/cl.html#2.3.1.0) Sp1 720 731

[2.3.1.0](http://transfac.gbf.de/TRANSFAC/cl/cl.html#2.3.1.0) Sp1 729 739

[2.3.2.1](http://transfac.gbf.de/TRANSFAC/cl/cl.html#2.3.2.1) Egr-1 744 753

[2.3.1.0](http://transfac.gbf.de/TRANSFAC/cl/cl.html#2.3.1.0) Sp1 744 754

[9.9.270](http://transfac.gbf.de/TRANSFAC/cl/cl.html#9.9.270) ETF 745 754

[9.9.537](http://transfac.gbf.de/TRANSFAC/cl/cl.html#9.9.537) NF-1 749 758

[2.3.1.0](http://transfac.gbf.de/TRANSFAC/cl/cl.html#2.3.1.0) Sp1 753 764

[2.3.1.0](http://transfac.gbf.de/TRANSFAC/cl/cl.html#2.3.1.0) Sp1 764 777

[2.3.1.0](http://transfac.gbf.de/TRANSFAC/cl/cl.html#2.3.1.0) Sp1 770 779

[9.9.561](http://transfac.gbf.de/TRANSFAC/cl/cl.html#9.9.561) NF-muE1 799 808

[4.3.2.0](http://transfac.gbf.de/TRANSFAC/cl/cl.html#4.3.2.0) SRF 802 811

[2.3.1.0](http://transfac.gbf.de/TRANSFAC/cl/cl.html#2.3.1.0) Sp1 804 818

[2.3.1.0](http://transfac.gbf.de/TRANSFAC/cl/cl.html#2.3.1.0) Sp1 811 821

[2.3.1.0](http://transfac.gbf.de/TRANSFAC/cl/cl.html#2.3.1.0) YY1 833 842

[2.3.1.0](http://transfac.gbf.de/TRANSFAC/cl/cl.html#2.3.1.0) Sp1 842 851

[2.3.1.0](http://transfac.gbf.de/TRANSFAC/cl/cl.html#2.3.1.0) Sp1 861 870

[3.5.1.2](http://transfac.gbf.de/TRANSFAC/cl/cl.html#3.5.1.2) REB1 862 871

[1.1.1.5](http://transfac.gbf.de/TRANSFAC/cl/cl.html#1.1.1.5) CPC1 867 876

[9.9.29](http://transfac.gbf.de/TRANSFAC/cl/cl.html#9.9.29) AP-1 867 877

[1.1.1.1](http://transfac.gbf.de/TRANSFAC/cl/cl.html#1.1.1.1) c-Jun 868 877

[9.9.32](http://transfac.gbf.de/TRANSFAC/cl/cl.html#9.9.32) AP-1 869 878

[2.3.1.0](http://transfac.gbf.de/TRANSFAC/cl/cl.html#2.3.1.0) Sp1 874 883

[3.5.1.2](http://transfac.gbf.de/TRANSFAC/cl/cl.html#3.5.1.2) RAP1 874 883

[4.3.2.0](http://transfac.gbf.de/TRANSFAC/cl/cl.html#4.3.2.0) SRF 885 894

[1.1.3.0](http://transfac.gbf.de/TRANSFAC/cl/cl.html#1.1.3.0) C/EBPalpha 903 912

[1.3.1.2](http://transfac.gbf.de/TRANSFAC/cl/cl.html#1.3.1.2) USF 919 928

[1.1.1.1](http://transfac.gbf.de/TRANSFAC/cl/cl.html#1.1.1.1) c-Jun 923 932

[1.1.2.0](http://transfac.gbf.de/TRANSFAC/cl/cl.html#1.1.2.0) ATF-1 924 933

[2.3.2.2](http://transfac.gbf.de/TRANSFAC/cl/cl.html#2.3.2.2) CF2-III 924 933

[2.3.1.0](http://transfac.gbf.de/TRANSFAC/cl/cl.html#2.3.1.0) Sp1 939 948

[2.3.3.0](http://transfac.gbf.de/TRANSFAC/cl/cl.html#2.3.3.0) MIG1 1000 1009

[2.3.1.0](http://transfac.gbf.de/TRANSFAC/cl/cl.html#2.3.1.0) Sp1 1001 1011

[9.9.588](http://transfac.gbf.de/TRANSFAC/cl/cl.html#9.9.588) NF-kappaB 1022 1031

[2.3.4.0](http://transfac.gbf.de/TRANSFAC/cl/cl.html#2.3.4.0) MBP-2 1023 1032

[4.1.1.0](http://transfac.gbf.de/TRANSFAC/cl/cl.html#4.1.1.0) c-Rel 1023 1032

[9.9.213](http://transfac.gbf.de/TRANSFAC/cl/cl.html#9.9.213) EBP-1 1023 1032

[9.9.590](http://transfac.gbf.de/TRANSFAC/cl/cl.html#9.9.590) NF-kappaB 1023 1032

[9.9.592](http://transfac.gbf.de/TRANSFAC/cl/cl.html#9.9.592) NF-kappaB(-like 1023 1032

[9.9.594](http://transfac.gbf.de/TRANSFAC/cl/cl.html#9.9.594) RelA 1023 1032

[3.5.1.2](http://transfac.gbf.de/TRANSFAC/cl/cl.html#3.5.1.2) RAP1 1038 1047

[9.9.77](http://transfac.gbf.de/TRANSFAC/cl/cl.html#9.9.77) CACCC-binding_f 1041 1050

[2.3.1.0](http://transfac.gbf.de/TRANSFAC/cl/cl.html#2.3.1.0) Sp1 1041 1051

[3.1.1.0](http://transfac.gbf.de/TRANSFAC/cl/cl.html#3.1.1.0) MATalpha2 1076 1085

[2.3.1.0](http://transfac.gbf.de/TRANSFAC/cl/cl.html#2.3.1.0) Sp1 1086 1095

[2.1.2.3](http://transfac.gbf.de/TRANSFAC/cl/cl.html#2.1.2.3) REV-ErbAalpha 1096 1105

[1.1.3.0](http://transfac.gbf.de/TRANSFAC/cl/cl.html#1.1.3.0) C/EBPalpha 1118 1127

[9.9.29](http://transfac.gbf.de/TRANSFAC/cl/cl.html#9.9.29) AP-1 1131 1140

[1.1.3.0](http://transfac.gbf.de/TRANSFAC/cl/cl.html#1.1.3.0) C/EBPgamma 1150 1159

[1.1.1.5](http://transfac.gbf.de/TRANSFAC/cl/cl.html#1.1.1.5) GCN4 1154 1163

[2.3.2.2](http://transfac.gbf.de/TRANSFAC/cl/cl.html#2.3.2.2) Hb 1155 1164

[4.3.2.0](http://transfac.gbf.de/TRANSFAC/cl/cl.html#4.3.2.0) SRF 1165 1174

**Table. 7 The TF binding sites predicted by AliBaba program for hPGK promoter.**

**Promoter: hPGK**

**Length: 516 bp**

**Number of sites found: 50**

Class Factor Start Stop

---------------------------------------------------

[1.1.3.0](http://transfac.gbf.de/TRANSFAC/cl/cl.html#1.1.3.0) C/EBPalpha 11 20

[1.1.3.0](http://transfac.gbf.de/TRANSFAC/cl/cl.html#1.1.3.0) C/EBPalpha 19 28

[2.3.1.0](http://transfac.gbf.de/TRANSFAC/cl/cl.html#2.3.1.0) Sp1 49 58

[2.3.1.0](http://transfac.gbf.de/TRANSFAC/cl/cl.html#2.3.1.0) Sp1 64 73

[9.9.1299](http://transfac.gbf.de/TRANSFAC/cl/cl.html#9.9.1299) MPBF 72 81

[3.5.1.2](http://transfac.gbf.de/TRANSFAC/cl/cl.html#3.5.1.2) FlbD 73 82

[2.3.1.0](http://transfac.gbf.de/TRANSFAC/cl/cl.html#2.3.1.0) Sp1 84 98

[2.3.1.0](http://transfac.gbf.de/TRANSFAC/cl/cl.html#2.3.1.0) Sp1 93 102

[1.1.1.6](http://transfac.gbf.de/TRANSFAC/cl/cl.html#1.1.1.6) CRE-BP1 132 141

[9.9.51](http://transfac.gbf.de/TRANSFAC/cl/cl.html#9.9.51) ATF 132 141

[2.3.3.0](http://transfac.gbf.de/TRANSFAC/cl/cl.html#2.3.3.0) CPE_binding_pro 133 142

[1.1.2.0](http://transfac.gbf.de/TRANSFAC/cl/cl.html#1.1.2.0) CREB 134 143

[3.5.1.2](http://transfac.gbf.de/TRANSFAC/cl/cl.html#3.5.1.2) REB1 135 144

[2.3.1.0](http://transfac.gbf.de/TRANSFAC/cl/cl.html#2.3.1.0) Sp1 162 174

[1.6.1.0](http://transfac.gbf.de/TRANSFAC/cl/cl.html#1.6.1.0) AP-2alphaA 166 175

[2.3.1.0](http://transfac.gbf.de/TRANSFAC/cl/cl.html#2.3.1.0) Sp1 168 181

[2.3.3.0](http://transfac.gbf.de/TRANSFAC/cl/cl.html#2.3.3.0) CPE_binding_pro 169 178

[3.5.2.0](http://transfac.gbf.de/TRANSFAC/cl/cl.html#3.5.2.0) c-Ets-1_68 177 186

[2.3.1.0](http://transfac.gbf.de/TRANSFAC/cl/cl.html#2.3.1.0) Sp1 179 188

[2.3.1.0](http://transfac.gbf.de/TRANSFAC/cl/cl.html#2.3.1.0) Sp1 185 195

[3.4.1.0](http://transfac.gbf.de/TRANSFAC/cl/cl.html#3.4.1.0) TSF3 202 211

[2.3.1.0](http://transfac.gbf.de/TRANSFAC/cl/cl.html#2.3.1.0) Sp1 221 235

[3.5.2.0](http://transfac.gbf.de/TRANSFAC/cl/cl.html#3.5.2.0) GABP 241 250

[1.1.1.6](http://transfac.gbf.de/TRANSFAC/cl/cl.html#1.1.1.6) CRE-BP1 251 260

[2.3.1.0](http://transfac.gbf.de/TRANSFAC/cl/cl.html#2.3.1.0) YY1 285 294

[1.1.3.0](http://transfac.gbf.de/TRANSFAC/cl/cl.html#1.1.3.0) C/EBPalpha 288 297

[3.5.1.2](http://transfac.gbf.de/TRANSFAC/cl/cl.html#3.5.1.2) Adf-1 295 304

[2.3.1.0](http://transfac.gbf.de/TRANSFAC/cl/cl.html#2.3.1.0) Sp1 298 312

[2.3.1.0](http://transfac.gbf.de/TRANSFAC/cl/cl.html#2.3.1.0) Sp1 305 314

[2.3.1.0](http://transfac.gbf.de/TRANSFAC/cl/cl.html#2.3.1.0) Sp1 314 327

[9.9.150](http://transfac.gbf.de/TRANSFAC/cl/cl.html#9.9.150) CP1 323 332

[9.9.539](http://transfac.gbf.de/TRANSFAC/cl/cl.html#9.9.539) NF-1 324 333

[3.5.1.2](http://transfac.gbf.de/TRANSFAC/cl/cl.html#3.5.1.2) Adf-1 331 340

[2.3.1.0](http://transfac.gbf.de/TRANSFAC/cl/cl.html#2.3.1.0) Sp1 342 354

[2.3.1.0](http://transfac.gbf.de/TRANSFAC/cl/cl.html#2.3.1.0) Sp1 361 372

[2.3.1.0](http://transfac.gbf.de/TRANSFAC/cl/cl.html#2.3.1.0) Sp1 369 383

[2.3.1.0](http://transfac.gbf.de/TRANSFAC/cl/cl.html#2.3.1.0) Sp1 377 389

[2.3.1.0](http://transfac.gbf.de/TRANSFAC/cl/cl.html#2.3.1.0) Sp1 384 397

[2.3.1.0](http://transfac.gbf.de/TRANSFAC/cl/cl.html#2.3.1.0) Sp1 392 404

[2.3.1.0](http://transfac.gbf.de/TRANSFAC/cl/cl.html#2.3.1.0) Sp1 398 407

[2.2.1.1](http://transfac.gbf.de/TRANSFAC/cl/cl.html#2.2.1.1) GATA-1 410 419

[2.3.1.0](http://transfac.gbf.de/TRANSFAC/cl/cl.html#2.3.1.0) Sp1 420 430

[2.3.1.0](http://transfac.gbf.de/TRANSFAC/cl/cl.html#2.3.1.0) Sp1 426 435

[3.6.1.0](http://transfac.gbf.de/TRANSFAC/cl/cl.html#3.6.1.0) TEC1 437 446

[2.3.1.0](http://transfac.gbf.de/TRANSFAC/cl/cl.html#2.3.1.0) Sp1 452 461

[1.3.1.2](http://transfac.gbf.de/TRANSFAC/cl/cl.html#1.3.1.2) USF 462 471

[2.3.1.0](http://transfac.gbf.de/TRANSFAC/cl/cl.html#2.3.1.0) Sp1 503 512

[1.6.1.0](http://transfac.gbf.de/TRANSFAC/cl/cl.html#1.6.1.0) AP-2 505 514

[2.2.1.1](http://transfac.gbf.de/TRANSFAC/cl/cl.html#2.2.1.1) GATA-1 505 514

[2.3.3.0](http://transfac.gbf.de/TRANSFAC/cl/cl.html#2.3.3.0) MIG1 509 518

**Table. 8 The TF binding sites predicted by AliBaba program for RPBSA promoter.**

**Promoter: RPBSA**

**Length: 612 bp**

**Number of sites found: 59**

Class Factor Start Stop

---------------------------------------------------

[1.1.3.0](http://transfac.gbf.de/TRANSFAC/cl/cl.html#1.1.3.0) C/EBPalpha 14 23

[2.3.1.0](http://transfac.gbf.de/TRANSFAC/cl/cl.html#2.3.1.0) Sp1 28 41

[2.3.1.0](http://transfac.gbf.de/TRANSFAC/cl/cl.html#2.3.1.0) Sp1 48 57

[1.6.1.0](http://transfac.gbf.de/TRANSFAC/cl/cl.html#1.6.1.0) AP-2alphaA 66 75

[2.3.1.0](http://transfac.gbf.de/TRANSFAC/cl/cl.html#2.3.1.0) Sp1 73 82

[2.3.1.0](http://transfac.gbf.de/TRANSFAC/cl/cl.html#2.3.1.0) Sp1 88 99

[1.6.1.0](http://transfac.gbf.de/TRANSFAC/cl/cl.html#1.6.1.0) AP-2alphaA 93 102

[3.5.1.2](http://transfac.gbf.de/TRANSFAC/cl/cl.html#3.5.1.2) REB1 117 126

[1.2.8.0](http://transfac.gbf.de/TRANSFAC/cl/cl.html#1.2.8.0) Id3 138 147

[2.3.1.0](http://transfac.gbf.de/TRANSFAC/cl/cl.html#2.3.1.0) Sp1 138 147

[2.2.1.1](http://transfac.gbf.de/TRANSFAC/cl/cl.html#2.2.1.1) GATA-3 166 175

[2.3.1.0](http://transfac.gbf.de/TRANSFAC/cl/cl.html#2.3.1.0) Sp1 172 181

[4.3.2.0](http://transfac.gbf.de/TRANSFAC/cl/cl.html#4.3.2.0) SRF 183 192

[2.3.1.0](http://transfac.gbf.de/TRANSFAC/cl/cl.html#2.3.1.0) Sp1 194 203

[2.3.1.0](http://transfac.gbf.de/TRANSFAC/cl/cl.html#2.3.1.0) Sp1 205 217

[1.1.3.0](http://transfac.gbf.de/TRANSFAC/cl/cl.html#1.1.3.0) C/EBPalpha 211 220

[1.6.1.0](http://transfac.gbf.de/TRANSFAC/cl/cl.html#1.6.1.0) AP-2alphaA 213 222

[2.3.1.0](http://transfac.gbf.de/TRANSFAC/cl/cl.html#2.3.1.0) Sp1 223 234

[2.3.2.1](http://transfac.gbf.de/TRANSFAC/cl/cl.html#2.3.2.1) Krox-20 225 234

[2.3.1.0](http://transfac.gbf.de/TRANSFAC/cl/cl.html#2.3.1.0) Sp1 229 242

[1.3.1.2](http://transfac.gbf.de/TRANSFAC/cl/cl.html#1.3.1.2) USF 237 246

[1.1.1.6](http://transfac.gbf.de/TRANSFAC/cl/cl.html#1.1.1.6) ATF-a 240 249

[1.1.2.0](http://transfac.gbf.de/TRANSFAC/cl/cl.html#1.1.2.0) CREB 240 249

[2.3.3.0](http://transfac.gbf.de/TRANSFAC/cl/cl.html#2.3.3.0) CPE_binding_pro 240 249

[2.3.1.0](http://transfac.gbf.de/TRANSFAC/cl/cl.html#2.3.1.0) Sp1 270 279

[3.5.1.2](http://transfac.gbf.de/TRANSFAC/cl/cl.html#3.5.1.2) RAP1 291 300

[1.1.3.0](http://transfac.gbf.de/TRANSFAC/cl/cl.html#1.1.3.0) C/EBPdelta 297 306

[2.3.1.0](http://transfac.gbf.de/TRANSFAC/cl/cl.html#2.3.1.0) Sp1 310 319

[2.3.1.0](http://transfac.gbf.de/TRANSFAC/cl/cl.html#2.3.1.0) Sp1 316 325

[2.3.4.0](http://transfac.gbf.de/TRANSFAC/cl/cl.html#2.3.4.0) MBP-2 331 340

[4.1.1.0](http://transfac.gbf.de/TRANSFAC/cl/cl.html#4.1.1.0) c-Rel 331 340

[9.9.213](http://transfac.gbf.de/TRANSFAC/cl/cl.html#9.9.213) EBP-1 331 340

[9.9.590](http://transfac.gbf.de/TRANSFAC/cl/cl.html#9.9.590) NF-kappaB 331 340

[9.9.594](http://transfac.gbf.de/TRANSFAC/cl/cl.html#9.9.594) RelA 331 340

[9.9.637](http://transfac.gbf.de/TRANSFAC/cl/cl.html#9.9.637) NRF-1 341 350

[9.9.1197](http://transfac.gbf.de/TRANSFAC/cl/cl.html#9.9.1197) NRF-1 341 350

[9.9.150](http://transfac.gbf.de/TRANSFAC/cl/cl.html#9.9.150) CP1 359 368

[3.5.2.0](http://transfac.gbf.de/TRANSFAC/cl/cl.html#3.5.2.0) c-Ets-1_68 374 383

[2.3.1.0](http://transfac.gbf.de/TRANSFAC/cl/cl.html#2.3.1.0) Sp1 383 392

[1.1.1.6](http://transfac.gbf.de/TRANSFAC/cl/cl.html#1.1.1.6) CRE-BP1 398 407

[1.1.3.0](http://transfac.gbf.de/TRANSFAC/cl/cl.html#1.1.3.0) C/EBPalpha 398 407

[2.3.3.0](http://transfac.gbf.de/TRANSFAC/cl/cl.html#2.3.3.0) CPE_binding_pro 398 407

[9.9.29](http://transfac.gbf.de/TRANSFAC/cl/cl.html#9.9.29) AP-1 400 409

[2.3.1.0](http://transfac.gbf.de/TRANSFAC/cl/cl.html#2.3.1.0) Sp1 411 420

[4.5.1.0](http://transfac.gbf.de/TRANSFAC/cl/cl.html#4.5.1.0) TBP 427 436

[4.4.1.0](http://transfac.gbf.de/TRANSFAC/cl/cl.html#4.4.1.0) E2 437 446

[3.4.1.0](http://transfac.gbf.de/TRANSFAC/cl/cl.html#3.4.1.0) HSTF 446 455

[2.3.1.0](http://transfac.gbf.de/TRANSFAC/cl/cl.html#2.3.1.0) YY1 473 482

[2.3.2.2](http://transfac.gbf.de/TRANSFAC/cl/cl.html#2.3.2.2) Hb 474 483

[2.3.2.2](http://transfac.gbf.de/TRANSFAC/cl/cl.html#2.3.2.2) Hb 492 501

[3.5.3.0](http://transfac.gbf.de/TRANSFAC/cl/cl.html#3.5.3.0) NF-EM5 496 505

[3.5.2.0](http://transfac.gbf.de/TRANSFAC/cl/cl.html#3.5.2.0) PU.1 499 508

[4.1.1.0](http://transfac.gbf.de/TRANSFAC/cl/cl.html#4.1.1.0) NF-kappaB 510 519

[9.9.539](http://transfac.gbf.de/TRANSFAC/cl/cl.html#9.9.539) NF-1 522 531

[2.1.2.10](http://transfac.gbf.de/TRANSFAC/cl/cl.html#2.1.2.10) COUP 535 544

[1.1.3.0](http://transfac.gbf.de/TRANSFAC/cl/cl.html#1.1.3.0) C/EBPalpha 565 574

[2.2.1.1](http://transfac.gbf.de/TRANSFAC/cl/cl.html#2.2.1.1) GATA-1 583 592

[9.9.701](http://transfac.gbf.de/TRANSFAC/cl/cl.html#9.9.701) PTF1-beta 587 596

[3.4.1.0](http://transfac.gbf.de/TRANSFAC/cl/cl.html#3.4.1.0) HSE-binding_pro 600 609

**Table. 9 The TF binding sites predicted by PROMO program.**

| **EF1**  **(67 TFBDs)** | **CMV**  **(62 TFBDs)** | **hPGK**  **(55 TFBDs)** | **RPBSA**  **(58 TFBDs)** |
| --- | --- | --- | --- |
| Pax-5 | C/EBPbeta | C/EBPbeta | RXR-alpha |
| p53 | C/EBPalpha | NF-1 | TFIID |
| c-Jun | GR-beta | TFII-I | HNF-3alpha |
| Egr-3 | HOXD9 | STAT4 | GR |
| ETF | HOXD10 | NF-AT1 | GR-beta |
| TFII-I | c-Jun | c-Ets-1 | Pax-5 |
| C/EBPbeta | Pax-5 | STAT1beta | p53 |
| NF-1 | p53 | GR-alpha | IRF-1 |
| GR-alpha | YY1 | AP-2alphaA | GR-alpha |
| GR-beta | SRF | NFI/CTF | AP-2alphaA |
| C/EBPalpha | TFII-I | Pax-5 | NF-AT1 |
| VDR | STAT4 | p53 | ENKTF-1 |
| PXR-1:RXR-alpha | E2F-1 | EBF | TFII-I |
| ENKTF-1 | c-Ets-1 | IRF-1 | YY1 |
| FOXP3 | HNF-1C | FOXP3 | XBP-1 |
| c-Myb | FOXP3 | NF-AT1 | C/EBPbeta |
| IRF-1 | HNF-1B | GCF | STAT4 |
| TFIID | c-Myb | RXR-alpha | c-Ets-1 |
| NF-AT1 | XBP-1 | c-Jun | Elk-1 |
| IRF-2 | ENKTF-1 | T3R-beta1 | FOXP3 |
| XBP-1 | ER-alpha | E2F-1 | PR B |
| E2F-1 | Sp1 | Elk-1 | PR A |
| NF-AT2 | NFI/CTF | Sp1 | VDR |
| STAT4 | RXR-alpha | TFIID | PXR-1:RXR-alpha |
| c-Ets-1 | ETF | HIF-1 | MEF-2A |
| PR B | ATF-1 | GR-beta | E2F-1 |
| PR A | CREB | XBP-1 | PPAR-alpha:RXR-alpha |
| RelA | ATF3 | ENKTF-1 | WT1 |
| Elk-1 | ATF-2 | ER-alpha | GCF |
| AhR:Arnt | ATF | ATF3 | c-Myc |
| AP-2alphaA | NF-kappaB | PR B | USF1 |
| YY1 | RBP-Jkappa | PR A | ER-alpha |
| HNF-3alpha | GR-alpha | c-Ets-2 | NFI/CTF |
| ER-alpha | NF-AT2 | ETF | AR |
| GATA-3 | NF-AT1 | C/EBPalpha | C/EBPalpha |
| NFI/CTF | NF-1 | CTF | NF-Y |
| PPAR-alpha:RXR-alpha | GATA-1 | NF-Y | NF-1 |
| GCF | STAT1beta | YY1 | ETF |
| GATA-1 | AP-1 | AR | AhR:Arnt |
| Sp1 | HNF-3alpha | AhR:Arnt | c-Ets-2 |
| GR | GR | NF-kappaB | c-Jun |
| AP-1 | TFIID | PPAR-alpha:RXR-alpha | Sp1 |
| RXR-alpha | PR B | RelA | c-Myb |
| c-Fos | PR A | GR | HNF-1C |
| T3R-beta1 | AP-2alphaA | RAR-beta | HNF-1B |
| SRY | RAR-beta |  | NF-AT2 |
| TCF-4E | Egr-3 |  | STAT1beta |
| c-Ets-2 | AhR:Arnt |  | COUP-TF1 |
| STAT1beta | POU2F1 |  |  |
| ATF3 | IRF-1 |  |  |
| NF-kappaB | NF-AT1 |  |  |
| NF-kappaB1 | NF-kappaB1 |  |  |
| HNF-4alpha | c-Fos |  |  |
| SRF | GATA-2 |  |  |
| LEF-1 | T3R-beta1 |  |  |
| TCF-4 | c-Ets-2 |  |  |
| RAR-beta | CTF |  |  |
| MAZ | NF-Y |  |  |
| AhR | E2F |  |  |
| EBF | VDR |  |  |
| HOXD9 | PXR-1:RXR-alpha |  |  |
| HOXD10 | RAR-alpha1 |  |  |
| STAT5A |  |  |  |
| Ik-1 |  |  |  |
| ATF-1 |  |  |  |
| E2F |  |  |  |
| RBP-Jkappa |  |  |  |
